# Occurrence of oxazolidinone resistance genes in enterococci, staphylococci, and *Mammaliicoccus sciuri* from swine slaughterhouse wastewater, Italy

**DOI:** 10.1007/s11274-026-04968-0

**Published:** 2026-04-18

**Authors:** Francesca Romana Massacci, Serena Simoni, Elisa Albini, Maria Elena Nigro, Elisa Russo, Gloria D’achille, Claudia Paoletti, Gianluca Morroni, Marina Mingoia, Yao Zhu, Wanjiang Zhang, Carla Vignaroli, Chiara Francesca Magistrali, Eleonora Giovanetti, Andrea Brenciani

**Affiliations:** 1 Department of Research and Development, Istituto Zooprofilattico Sperimentale dell’Umbria e delle Marche ‘Togo Rosati’, Perugia, Italy; 2 Unit of Microbiology, Department of Life and Environmental Sciences, Polytechnic University of Marche, Ancona, Italy; 3 Unit of Microbiology, Department of Biomedical Sciences and Public Health, Polytechnic University of Marche Medical School, Via Tronto 10/A, Ancona, 60126 Italy; 4 SOS Microbiology, SOD Laboratory Medicine, Azienda Ospedaliero Universitaria delle Marche, Ancona, Italy; 5 State Key Laboratory for Animal Disease Control and Prevention, Harbin Veterinary Research Institute, Chinese Academy of Agricultural Sciences, Harbin, 150069 China; 6 Department of Sede Territoriale Lodi-Milano, Istituto Zooprofilattico Sperimentale Della Lombardia e Dell’Emilia Romagna ‘Bruno Ubertini’, Brescia, 25124 Italy

**Keywords:** *Enterococcus* spp., *Staphylococcus* spp., *Mammaliicoccus sciuri*, slaughterhouse wastewater, *cfr*, *optrA*, *poxtA*

## Abstract

Oxazolidinones (linezolid and tedizolid) are last-resort antibiotics used to treat severe infections caused by multidrug-resistant Gram-positive bacteria. Although linezolid is not approved for veterinary use, resistant bacteria—particularly enterococci—have increasingly been reported in food-producing animals worldwide. Slaughterhouses represent potential hotspots for the transmission of antimicrobial-resistant pathogens along the food chain. This study investigated the presence of oxazolidinone resistance genes and relevant genetic elements in florfenicol-resistant enterococci and staphylococci isolated from wastewater collected from an Italian swine slaughterhouse. In total, five enterococci, six staphylococci, and one *Mammaliicoccus sciuri* (previously known as *Staphylococcus sciuri*) isolate were recovered. PCR screening revealed that all staphylococci and *M. sciuri* carried the *cfr* gene, while enterococci were positive for either *optrA* or *poxtA*. Nine isolates were selected for whole genome sequencing. *Enterococcus hirae* EM2 carried *poxtA* on the novel pEhEM2-*poxtA* plasmid. In *E. faecium* ED1 and EF1, *optrA* was located on the novel pEfmED1-*optrA* plasmid. *E. durans* EF2 carried *poxtA* within a Tn*6657* transposon integrated into a pEgFS4-2-like plasmid also containing *tet*(M), *tet*(L), and genes related to transposition and plasmid replication. In *Staphylococcus simulans* SN1 and SF3, *cfr* was found on the novel pSsSN1-*cfr* plasmid and on p12-02300 plasmid, respectively. In *Staphylococcus cohnii* SD1, *cfr* was located on the previously described plasmid unnamed1, while in *S. cohnii* SF2 it was inserted into a chromosomal Tn*558*-like transposon along with *fexA*. *M. sciuri* SN4 harboured *cfr* on a small plasmid showing high similarity to the pK8D55P-*cfr* plasmid previously found in an *M. sciuri* isolate from ducks. These results highlight the role of slaughterhouse wastewater as a potential reservoir of oxazolidinone resistance genes and emphasize the need for continued monitoring to limit the environmental spread of antimicrobial resistance from animal production systems.

**Graphical Abstract:**

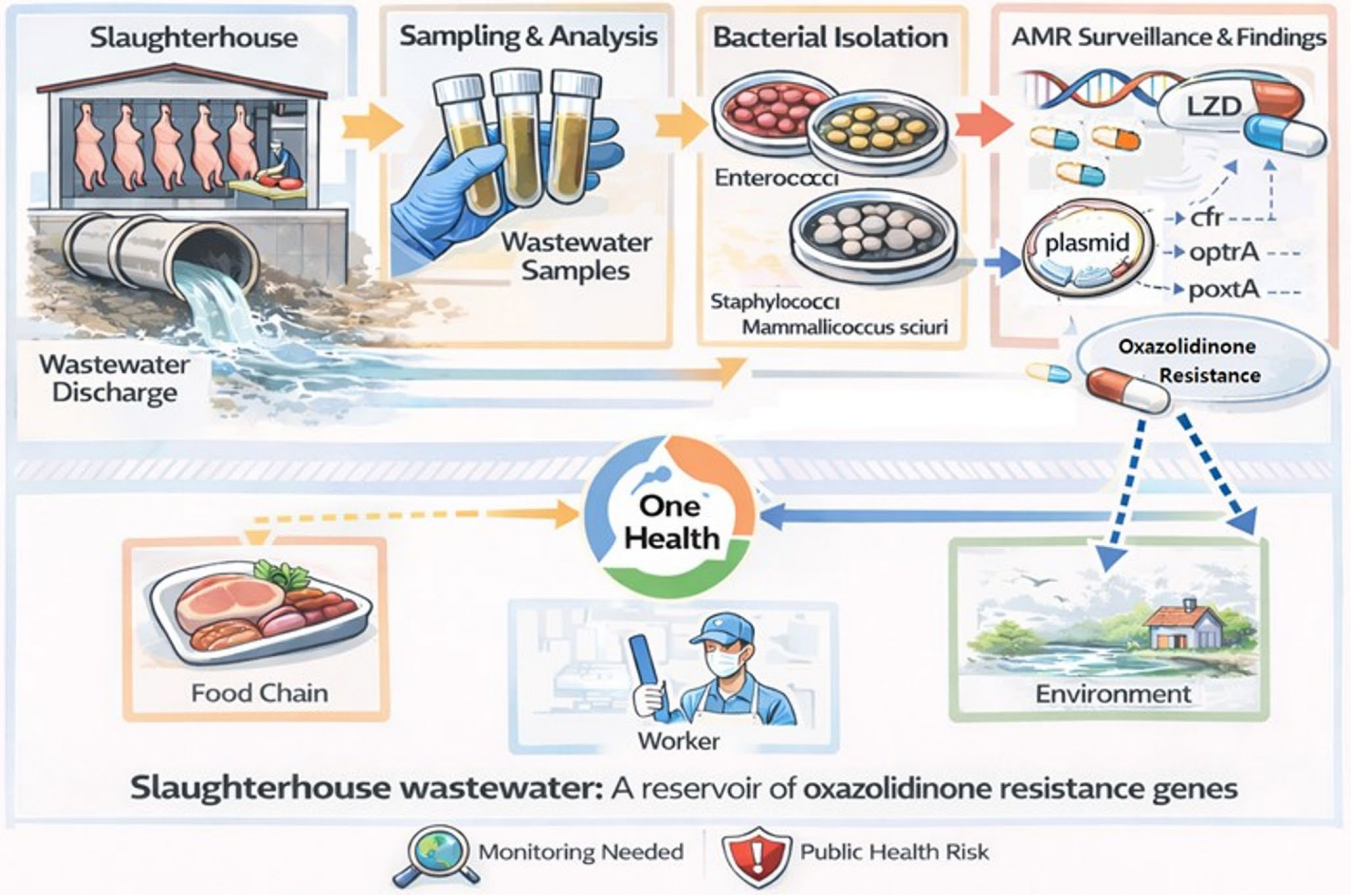

**Supplementary Information:**

The online version contains supplementary material available at 10.1007/s11274-026-04968-0.

## Introduction

The overuse of antibiotics in animal husbandry for both disease prevention and treatment is widely recognized as a major driver of antimicrobial resistance emergence in livestock-associated bacteria worldwide. This misuse of antimicrobials has facilitated not only the spread of resistance to veterinary drugs, but also the emergence of new resistance mechanisms, and the development of cross-resistance to antibiotics exclusively for human use (Marshall et al. 2011; Tang et al. 2017). Livestock can therefore act as reservoirs of antibiotic-resistant pathogens transmissible to humans, both through direct contact and via cross-contamination of food products, thereby contributing to the spread of resistant bacteria along the food chain (Pandey et al. 2024). In addition, animals contribute to environmental pollution by excreting unmetabolized antimicrobial residues and by spreading antibiotic-resistant bacteria, especially through the contamination of surface waters (Barathe et al. 2024). Slaughterhouse wastewater is also considered a source of clinically relevant antibiotic-resistant bacteria and resistance genes that conventional treatment methods cannot fully eliminate (Savin et al. 2020a, b, 2022; Cong et al. 2023).

Foyle et al. provided a critical assessment of antibiotic resistance patterns in wastewater effluents from food animal slaughter facilities via a systematic evaluation of existing literature (Foyle et al. 2023). This review highlights that anthropogenic practices in intensive livestock farming have significantly contributed to the environmental dissemination of antimicrobial resistance through slaughterhouse wastewater, with resistance reported across all major antimicrobial classes — including some last-resort antibiotics, particularly ciprofloxacin (Foyle et al. 2023; Simonetti et al. 2012, 2020).

The oxazolidinones (linezolid and tedizolid) are last-resort antibiotics used to treat severe human infections due to multidrug-resistant Gram-positive bacteria (Schwarz et al. 2021; Brenciani et al. 2022). Resistance to linezolid can arise via ribosomal mutations, but also through the acquisition of transferable resistance genes: *cfr* and its variants, *optrA*, *poxtA* and *poxtA2* (Schwarz et al. 2021; Brenciani et al. 2022). *cfr* and *cfr*-like genes encode rRNA methyltransferases that catalyse the post-transcriptional methylation of 23 S rRNA resulting in a co-resistance to phenicols, lincosamides, oxazolidinones, pleuromutilins, and streptogramins group A (PhLOPS_A_ phenotype). *optrA* and *poxtA/poxtA2* genes encode ABC-F proteins leading to a decreased susceptibility to phenicols, and oxazolidinones (including tedizolid) (Schwarz et al. 2021; Brenciani et al. 2022).

Although oxazolidinones have been approved exclusively for human use (European Medicine Agency 2025), an increasing number of linezolid-resistant bacteria — particularly enterococci — has been detected in food-producing animals and wildlife (Fioriti et al. 2020; Torres et al. 2018; Nüesch-Inderbinen et al. 2023; Cinthi et al. 2024; Albini et al. 2023) as well as in the environmental setting (Fioriti et al. 2021a, b; Biggel et al. 2021; Cinthi et al. 2022a, b).

Several studies have examined wastewater effluents from animal slaughterhouses which are considered as a hotspot for antibiotic-resistant bacteria; however, only a few have specifically investigated the occurrence of linezolid-resistant enterococci and staphylococci (Losito et al. 2005; Savin et al. 2020a, b; Muwonge et al. 2025; Hasanpour et al. 2025) and none of them detected their presence.

It is now well established that the selective pressure imposed by the extensive use of antimicrobial agents in veterinary medicine — particularly phenicols — has promoted the spread of linezolid resistance genes (Schwarz et al. 2021; Holman et al. 2024). These genes may be transferred to human pathogens, posing a significant concern for public health (Schwarz et al. 2021; Brenciani et al. 2022).

To our knowledge, this study represents the first investigation concerning the occurrence of enterococci and staphylococci carrying linezolid resistance genes in wastewater samples recovered from a swine slaughterhouse by an in-depth genomic analysis.

## Materials and methods

### Sampling procedures and strains isolation

The study was conducted in one of the largest slaughterhouses in Italy, located in central Italy, with a processing capacity of approximately 2,500 pigs per day. Animals were sourced from domestic farms, providing a representative sample of the Italian pig population.

Four aliquots of wastewater were collected at the same sampling point in November, December 2023, February and March 2024. One hundred mL from each sample were filtered through a 0.45 μm Millipore membrane filter. The membrane was then vortexed in physiological saline, and 100 µL of the recovered bacteria were spread on Slanetz-Bartley and Chapman (Oxoid, Basingstoke, UK) agar plates supplemented with florfenicol (10 mg/L) for the selection of resistant enterococci and staphylococci, respectively.

### Genotypic and phenotypic characterization and typing assays

Identification of isolates was carried out by MALDI-TOF MS (Microflex LT Smart Biotyper with FlexControlBiotyper 3.4 software, Bruker Daltonics, Bremen, Germany). Florfenicol-resistant enterococci and staphylococci were screened by PCR for the presence of *cfr*, *cfr*-like, *optrA* and *poxtA* genes using primer pairs previously described (Cinthi et al. 2022a, b). Isolates with at least one linezolid resistance gene, were tested for their susceptibility to florfenicol, chloramphenicol, linezolid, vancomycin, erythromycin, tetracycline, oxacillin (SigmaAldrich, St. Louis, MI, USA) and tedizolid (Thermo Fisher Scientific, Waltham, USA) by standard broth microdilution assay. The susceptibility of enterococci to oxacillin was not tested. Susceptibility tests were interpreted according to CLSI clinical breakpoints (https://em100.edaptivedocs.net/GetDoc.aspx?doc=CLSI%20M100%20ED35:2025&scope=user). Since no CLSI breakpoint is currently available for florfenicol, a MIC value of 10 mg/L was adopted as the resistance breakpoint. *E. faecalis* ATCC 29,212 and *Staphylococcus aureus* ATCC 29,213 were used as quality controls.

Typing was performed by SmaI- pulsed-field gel electrophoresis (PFGE) as previously described (Ripa et al. 2001).

### WGS and sequence analysis

Bacterial genomic DNA was extracted by the QIAcube automated extractor using DNeasy PowerLyzer PowerSoil Kit according to the manufacturer’s instructions (Qiagen, Germany). Extracted DNA was subjected to whole-genome sequencing (WGS) using both the short-read Illumina MiSeq platform (Illumina Inc., San Diego, CA, USA) with a 2 × 150 bp paired end and a long- read MinION, Oxford Nanopore Technologies sequencing approach (MicrobesNG, Birmingham, UK). Unicycler v. 0.4.8 software was used for the hybrid assembly of short and long reads (https://github.com/rrwick/Unicycler). The RAST tool (https://rast.nmpdr.org/).

was used for the annotation of DNA sequences.

In silico identification of acquired antimicrobial resistance genes, molecular typing and phylogenetic analysis were carried out using dedicated tools available at the Center for Genomic Epidemiology (available at http://www.genomicepidemiology.org/) (ResFinder v.3.2, LRE-Finder 1.0, MLST 2.0, VirulenceFinder 2.0, CSI Phylogeny 1.4 to determine the single nucleotide polymorphism (SNP) differences between some strains of the study) using default parameters, and by the BLAST site (https://blast.ncbi.nlm.nih.gov/Blast.cgi). The BRIG software package, version 0.95, was used to create and to compare plasmids. The Easyfig tool was used to compare relevant genetic elements (https://mjsull.github.io/Easyfig/).

### Genomic data availability

The WGS data of *E. faecium* ED1 and EF1, *S. simulans* SN1 and SF3, *S. cohnii* SD1 and SF2, *E. durans* EF2, *E. hirae* EM2 and *M. sciuri* SN4 are available under the BioProject ID PRJNA1295837.

## Results

### Linezolid resistance genes, antibiotic susceptibility and typing

Overall, five florfenicol-resistant enterococci (3 *Enterococcus faecium*, 1 *Enterococcus hirae* and 1 *Enterococcus durans*), six florfenicol-resistant staphylococci (4 *Staphylococcus simulans* and 2 *Staphylococcus cohnii*) and one florfenicol-resistant *Mammaliicoccus sciuri* (previously known as *Staphylococcus sciuri*) were isolated from the four wastewater samples. PCR screening revealed the presence of the *cfr* gene in all staphylococci and in *M. sciuri*, while enterococcal isolates were positive either for the *optrA* or *poxtA* gene (Table 1).

**Table 1:** Isolation data, linezolid resistance genotype and typing of enterococci, staphylococci and *M. sciuri* isolate investigate in this study

Isolate	Month and year of sampling	*Linezolid resistance genes*			SmaI-PFGE Pulsotype
		*cfr*	*optrA*	*poxtA*	
*E. faecium* ED1	December 2023	-	+	-	B
*E. faecium* EF1	February 2024	-	+	-	A
*E. faecium* EM1	March 2024	-	+	-	A
*E. durans* EF2	February 2024	-	-	+	-
*E. hirae* EM2	March 2024	-	-	+	-
*S. simulans* SN1	November 2023	+	-	-	C
*S. simulans* SD3	December 2023	+	-	-	C
*S. simulans* SF1	February 2024	+	-	-	C1
*S. simulans* SF3	February 2024	+	-	-	D
*S. cohnii* SD1	December 2023	+	-	-	E
*S. cohnii* SF2	February 2024	+	-	-	F
*M. sciuri* SN4	November 2023	+	-	-	-

Isolates were all resistant to florfenicol (MIC range, 32–128 mg/L), chloramphenicol (MIC range, 32–128 mg/L), and either susceptible or resistant to linezolid (MIC range, 0.5–8 mg/L), tedizolid (MIC range, 0.5–2 mg/L), erythromycin (MIC range, 0.5–>128 mg/L), tetracycline (MIC range, 0.5–128 mg/L) and oxacillin (MIC range, 0.25–32 mg/L). All tested strains were susceptible to vancomycin (MIC range, 0.5–2 mg/L) (Table 2).

**Table 2:** Resistance profile of enterococci, staphylococci and *M. sciuri* isolate

Isolate	MIC (mg/L) of:							
	FFC	CHL	LZD	TZD	VAN	ERY	TET	OXA
*E. faecium* ED1	64	32	8	2	1	4	128	-^1^
*E. faecium* EF1	64	64	2	1	1	64	128	-
*E. faecium* EM1	128	32	8	2	0.5	8	64	-
*E. durans* EF2	32	32	2	0.5	0.5	> 128	128	-
*E. hirae* EM2	128	32	8	2	0.5	> 128	128	-
*S. simulans* SN1	> 128	128	8	1	1	> 128	128	0.25
*S. simulans* SD3	128	64	8	1	0.5	0.5	16	0.25
*S. simulans* SF1	> 128	128	16	2	1	1	1	32
*S. simulans* SF3	64	64	0.5	0.5	1	> 128	0.5	0.25
*S. cohnii* SD1	> 128	128	8	0.5	1	> 128	32	4
*S. cohnii* SF2	> 128	128	4	0.5	2	> 128	2	2
*M. sciuri* SN4	> 128	128	4	0.5	1	32	128	32

FFC, florfenicol; CHL, chloramphenicol; LZD, linezolid; TZD, tedizolid; VAN, vancomycin; ERY, erythromycin; TET, tetracycline; and OXA, oxacillin

MIC resistance breakpoints (CLSI) were as follows: florfenicol, not applicable (it is well known that a MIC value of ≥ 10 mg/L is to be considered within the resistance range); chloramphenicol, resistant at ≥ 32 mg/L; linezolid, resistant at ≥ 4 mg/L; tedizolid, susceptible at ≤ 0.5 mg/L (only for *E. faecalis*); tetracycline, resistant at ≥ 16 mg/L; vancomycin, resistant at ≥ 4 mg/L; oxacillin (only for *Staphylococcus* spp.), resistant at ≥ 1 mg/L

^1^The susceptibility of enterococci to oxacillin was not tested

SmaI-PFGE analysis revealed that *E. faecium* EF1 and *E. faecium* EM1 shared the same pattern (pulsotype A), as did *S. simulans* SN1 and *S. simulans* SD3 (pulsotype C), while *S. simulans* SF1 displayed a closely related pattern, designated C1 (Table 1).

### WGS analysis

Based on the typing data, four enterococci (*E. faecium* ED1, *E. faecium* EF1, *E. durans* EF2, and *E. hirae* EM2), four staphylococci (*S. simulans* SN1, S. *simulans* SF3, *S. cohnii* SD1, and *S. cohnii* SF2) and *M. sciuri* SN4, were selected for WGS analysis (only one strain per pulsotype, Table 1).

ResFinder analysis of the nine genomes revealed complex resistomes, with several acquired antibiotic resistance genes in addition to linezolid resistance determinants (Table 3). Genes responsible for the resistance to aminoglycosides, macrolides, lincosamides, phenicols and tetracyclines were detected in almost all isolates. Moreover, *E. faecium* strains exhibited two virulence genes encoding adhesins and surface proteins. No mutations involving the 23 S rRNA or L3/L4 ribosomal proteins were detected either in the assembled genomes or using LRE-finder software.

**Table 3:** Molecular typing, resistome and virulome of enterococci, staphylococci, and *M. sciuri* isolate investigated in this study

Isolate	ST	Resistome	Virulome
*E. faecium* ED1	ST1833	*aac*(6’)-*Ii*, *ant*(6)-*Ia*, *msr*(C), *erm*(A), *lsa*(E), *lnu*(B), *optrA*, *fexA*, *tet*(M), *tet*(L)	*acm*,* efaAfm*
*E. faecium* EF1	ST149	*aac*(6’)-*Ii*, *ant*(9)-*Ia*, *msr*(C), *erm*(A), *lnu*(A), *optrA*, *fexA*, *tet*(M), *tet*(L)	*efaAfm*
*E. durans* EF2	-	*aac*(6’)-*Ii*, *ant*(6)-*Ia*, *msr*(C), *erm*(A), *lsa*(E), *lnu*(B), *optrA*, *fexA*, *tet*(M), *tet*(L)	-
*E. hirae* EM2	-	*aac*(6’)-*Iid*, *ant*(6)-*Ia*, *lsa*(E), *lnu*(B), *erm*(B), *poxtA*, *optrA*, *fexB*, *fexA*, *tet*(M), *tet*(L)	-
*S. simulans* SN1	-	*aadD*, *vga*(A), *lsa*(B), *erm*(C), *vga*(A), *fexA*, *cfr*, *tet*(L), *dfrK*, *bleO*	-
*S. cohnii* SD1	-	*aac*(6’)-*aph*(2’’), *aadD*, *mecA*, *msr*(A), *erm*(C), *fexA*, *cfr*, *cat*(pC233), *tet*(K)	-
*S. cohnii* SF2	-	*msr*(A), *vga*(A), *erm*(C), *fexA*, *cfr*	-
*S. simulans* SF3	-	*str*, *vga*(A), *erm*(C), *fexA*, *cfr*	-
*M. sciuri* SN4	-	*aadD*, *aac*(6’)-*aph*(2’’), *mecA*, *mecA1*, *vga*(E), *sal*(A), *lnu*(A), *erm*(C), *fexA*, *cfr*, *tet*(L), *dfrD*	-

Subsequent bioinformatic analyses focused on the genetic contexts of the *optrA* and *poxtA* resistance genes in enterococci, and *cfr* gene in staphylococci and *M. sciuri*; their major features are summarized in the Table 4.

**Table 4:** Major features of genetic elements carrying linezolid resistance genes

Strain	Antibiotic resistance genes	OptrA protein variant	*cfr*, *optrA *and *poxtA *genetic contexts	Rep family plasmid	Genetic elements (accession no.)	Size (bp)	References
*E. faecium* ED1	*optrA*^1^, *fexA*,* erm*(A)	EDM^2^	Tn*6261*-like	ND^3^	pEfmED1-*optrA*	68,929	This study
*E. faecium* EF1	*optrA*^1^, *fexA*,* erm*(A)	DDD^2^	Tn*6261*-like	ND^3^	pEfmED1-*optrA*-like	63,909	This study
*E. durans* EF2	*poxtA*^1^, *fexB*,* tet*(M), *tet*(L)	-	Tn*6657*	Inc18	pEgFS4-2 (MZ291453)	38,387	Coccitto et al. 2022
*E. hirae* EM2	*poxtA*^1^, *fexB*,* lnu*(B), *lsa*(E), *ant(9)-I*, *ant(6)-I*,* tet*(M), *tet*(L)	-	Tn*6657*	Inc18, Rep_trans	pEhEM2-*poxtA*	83,274	This study
*E. hirae* EM2	*optrA*^1^, *fexA*	WT^4^	-	Rep3	pE349 (KP399637)	36,331	Wang et al. 2015
*S. simulans* SN1	*cfr*^1^, *fexA*,* lsa*(B), *ant(4’)-Ia*,* tet*(L), *dfrK*	-	-	Rep1	pSsSN1-*cfr*	54,959	This study
*S. simulans* SF3	*cfr*^1^, *fexA*	-	Tn*558*-like	ND^3^	p12-02300 (KM521837)	39,339	Bender et al. 2016
*S. cohnii* SD1	*cfr*^1^, *fexA*	-	Tn*558*-like	ND^3^	plasmid unnamed1 (CP126541)	39,805	Not published
*S. cohnii* SF2	*cfr*^1^, *fexA*	-	Tn*558*-like	-	Chromosomal region	9,718	This study
*M. sciuri* SN4	*cfr*^1^, *erm*(C)	-	-	Rep1	pMsSN4-*cfr*	6,183	This study

^1^Oxazolidinone resistance genes are indicated in bold

^2^Positions of OptrA protein mutations (in bold was indicated the mutated amino acid): *E. faecium* ED1 (K3**E**, Y176**D**, I622**M**) and *E. faecium* EF1 (G40**D**, Y176**D**, G393**D**)

^3^ND, not detectable

^4^WT, wild-type

### Enterococcal isolates

**Fig. 1:**
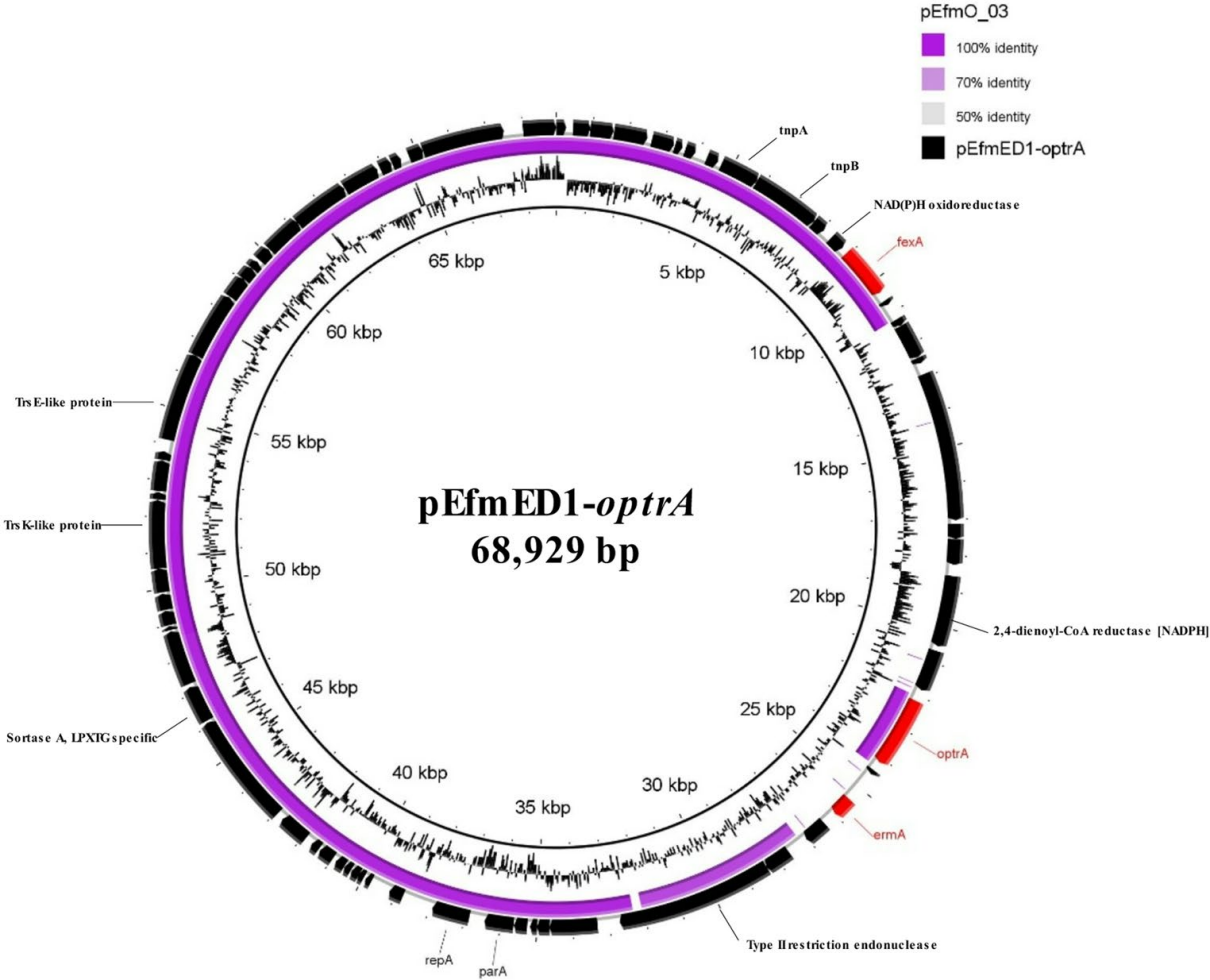
Circular map of the pEfmED1-*optrA* plasmid detected in both *E. faecium* ED1 and *E. faecium* EF1. The plasmid was aligned with the similar reported pEfmO_03 plasmid (accession n. MT261365) using BRIG software. Black arrows indicate the positions and orientations of genes; some antibiotic resistance determinants (red) and relevant genes described in this study are shown

In *E. faecium* ED1, the *optrA* gene was co-located with *fexA* on a novel 68,929-bp plasmid named pEfmED1-*optrA*, which did not belong to any known rep families. This plasmid shared 79% coverage and 99.60% nucleotide identity with the pEfmO_03 plasmid (58,684 bp, accession no. MT261365) previously identified in the human *E. faecium* O_03 strain from Ireland (Egan et al. 2020) (Fig. 1, Table S1). Compared to pEfmO_03 plasmid, pEfmED1-*optrA* showed an additional 10.8-kb region containing ORFs encoding hypothetical proteins, a DNA-binding protein and a 2,4-dienoyl-CoA reductase (NADPH) (Table S1). In this novel plasmid, the *optrA* gene was co-located with the *erm*(A) (macrolide, lincosamide, group B streptogramin resistance) within an 8,886-bp transposon, nearly identical (96% coverage, 99.31% nucleotide identity) to the reference sequence of Tn*6261* (accession no. KU354267) originally reported in a swine *E. faecalis* strain in China (Sun et al. 2018). *E. faecium* ED1 exhibited the EDM (K3E, Y176D, I622M) OptrA variant previously detected in human *E. faecalis* and *E. faecium* isolates (Morroni et al. 2017; Schwarz et al. 2021).

*E. faecium* EF1 harboured the *optrA* gene on a 63,909-bp plasmid showing 92% coverage and 98.95% nucleotide identity with the pEfmED1-*optrA* detected in *E. faecium* ED1 (see above). In *E. faecium* EF1, the DDD OptrA variant (G40D, Y176D, G393D) — previously reported in a *Streptococcus suis* of unknown origin (Schwarz et al. 2021) — was detected.

*E. faecium* ED1 and EF1 differed by 27,560 SNPs and belonged to ST1833 and ST149, respectively (Table 3), rare clones for which no information is currently available in either PubMed (https://pubmed.ncbi.nlm.nih.gov/) or PubMLST (https://pubmlst.org/).

*E. durans* EF2 harboured the *poxtA* gene on a 38,387-bp plasmid identical to plasmid pEgFS4-2 (accession no. MZ291453) (Inc18 replicon type) from *E. gallinarum* FS4 of swine origin, isolated in Italy (Coccitto et al. 2022). The *poxtA* gene was located within the Tn*6657* transposon also containing *fexB* (phenicol resistance), as originally described in the clinical MRSA AOUC-0915 (accession no. MH746818) (D’Andrea et al. 2019).

**Fig. 2:**
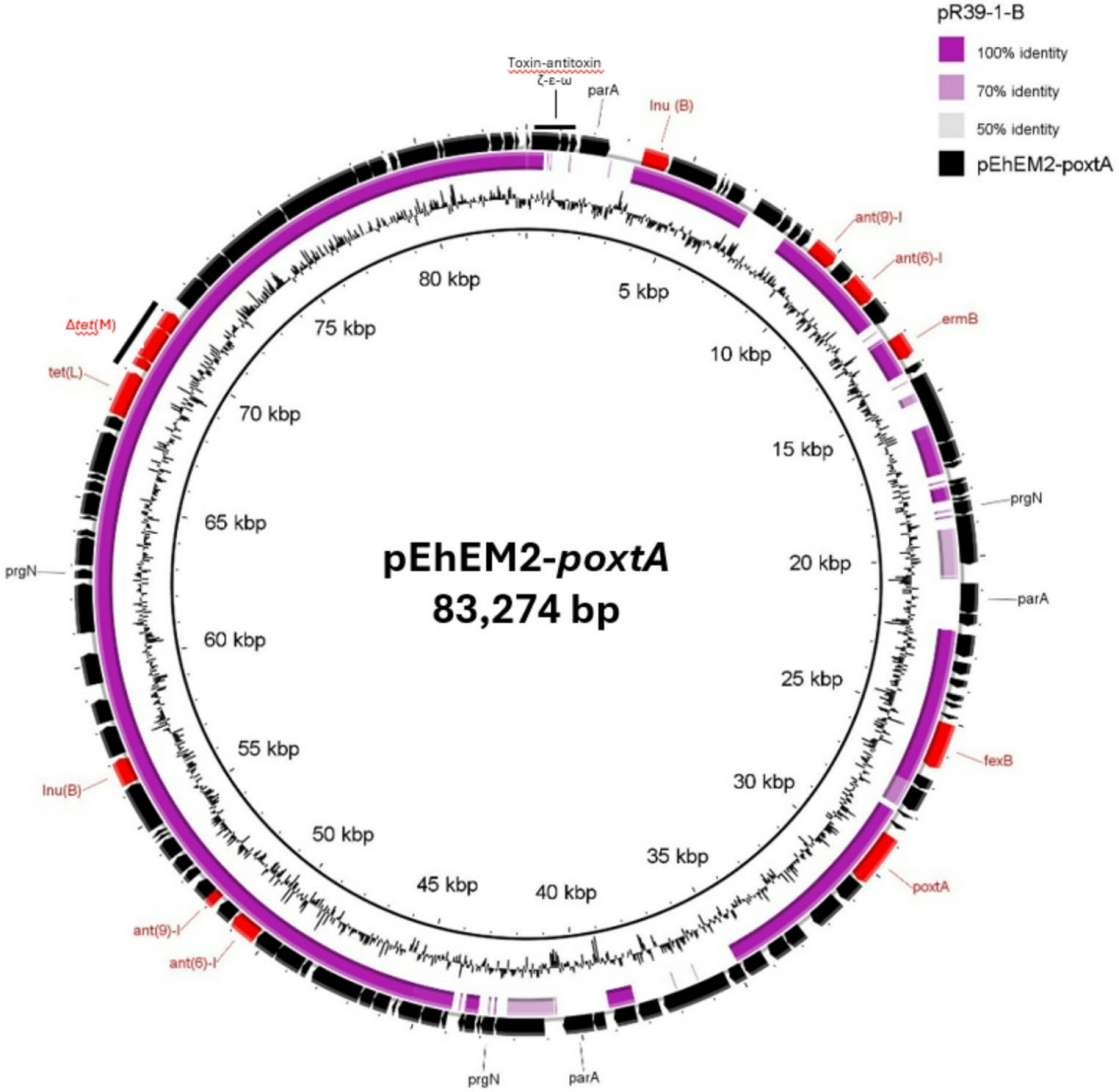
Circular map of the pEhEM2-*poxtA* plasmid detected in *E. hirae* EM2. The plasmid was aligned with the similar reported pR39-1-B plasmid (accession no. CP116519.1) using BRIG software. Black arrows indicate the positions and orientations of genes; some antibiotic resistance determinants (red) and relevant genes described in this study are shown

*E. hirae* EM2 carried the *poxtA* gene on a novel 83,274-bp plasmid, named pEhEM2-*poxtA*. The plasmid exhibited 81% coverage and 99.73% nucleotide identity to the pR39-1-B plasmid (77,238 bp, accession no. CP116519.1) previously identified in *E. faecium* R39-1 from bovine feces in Switzerland (Fig. 2, Table S2) (Nüesch-Inderbinen et al. 2023). In both plasmids, the *poxtA* context — flanked by two IS*1216* elements in the same orientation — was located within the Tn*6657* transposon also carrying the *fexB* gene (D’Andrea et al. 2019). The pEhEM2-*poxtA* plasmid belonged to Inc18 and Rep_trans rep families and carried genes encoding numerous transposases, plasmid partition proteins, an ω-ε-ζ antitoxin toxin system for plasmid stability within the bacterial population, an integrase/recombinase and several antibiotic resistance genes, in addition to *poxtA* and *fexB*, including *lnu*(B) (lincosamide resistance), *lsa*(E) (lincosamide and group A streptogramin resistance), *ant*(9)-I and *ant*(6)-I (aminoglycoside resistance), *tet*(L) and the truncated *tet*(M) (tetracycline resistance) (Fig. 2, Table S2).

*E. hirae* EM2 also carried the *optrA* gene co-located with *fexA* (phenicol resistance) on a 36,330-bp plasmid identical to the pE349 plasmid (36,331 bp, accession no. KP399637.1) (Rep3 replicon type) from the clinical *E. faecalis* E349 isolated in China (Wang et al. 2015). pE394 was the first *optrA*-carrying plasmid to be identified; accordingly, a wild-type OptrA protein was detected in *E. hirae* EM2.

#### Staphylococcal isolates

**Fig. 3:**
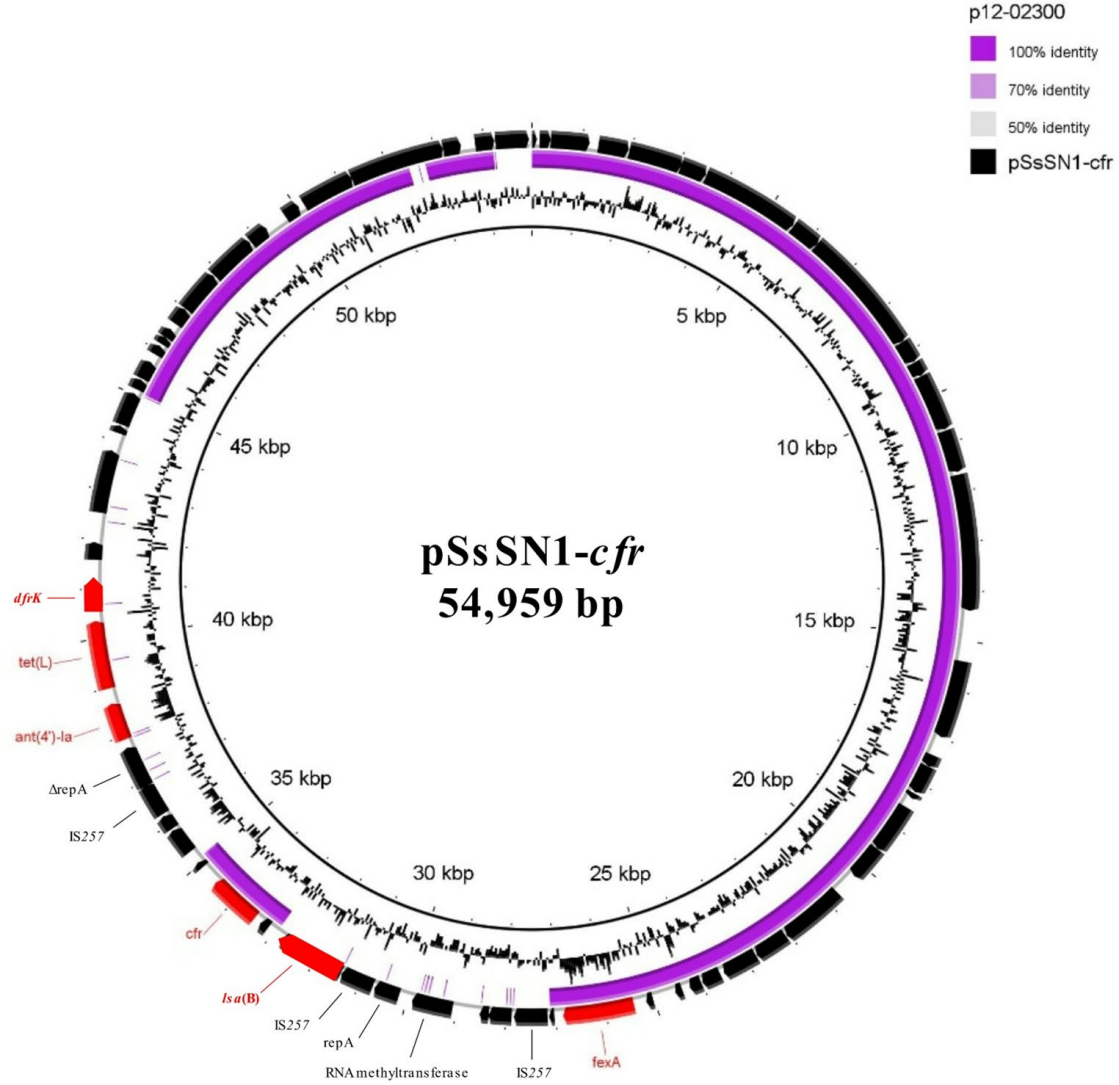
Circular map of the pSsSN1-*cfr* plasmid detected in *S. simulans* SN1. The plasmid was aligned with the similar reported p12-02300 plasmid (accession no. KM521837) using BRIG software. Black arrows indicate the positions and orientations of genes; some antibiotic resistance determinants (red) and relevant genes described in this study are shown

*S. simulans* SN1 carried the *cfr* and *fexA* genes on a novel 54,959-bp plasmid (Rep1 replicon type), named pSsSN1-*cfr*, which showed 98% coverage and 99.23% nucleotide identity with the smaller plasmid p12-02300 (38,864 bp, accession no. KM521837) from the clinical *S. epidermidis* 12-02300 strain isolated in Germany (Bender et al. 2015). Compared to p12-02300, the new plasmid pSsSN1-*cfr* exhibited two novel regions of 5,835 bp and 9,982 bp, respectively (Fig. 3, Table S3). The first region contained genes coding for IS*257* transposases, replication proteins, a putative RNA methyltransferase, and the *lsa*(B) gene (lincosamide resistance), while the second region included genes encoding a recombinase family protein, IS*257* transposases, replication proteins, MazG nucleotide pyrophosphohydrolase, MobV relaxase and three antibiotic resistance genes: *ant(4’)-Ia* (aminoglycoside resistance), *tet*(L) (tetracycline resistance), and *dfrK* (trimethoprim resistance) (Fig. 3, Table S3).

In *S. simulans* SF3, the *cfr* gene was located on a 39,339-bp plasmid, which did not belong to any known rep families, showing 100% coverage and 99.96% nucleotide identity with the p12-02300 plasmid (38,864 bp; accession no. KM521837), which did not belong to any known rep families, from the clinical *S. epidermidis* 12-02300 strain isolated in Germany (Bender et al. 2015). In this plasmid, *cfr* was located within the Tn*558* transposon that also carries the *fexA* gene (Kehrenberg et al. 2005). Interestingly, despite the presence of *cfr* gene, *S. simulans* SF3 exhibited susceptibility to linezolid (Table 2). Consequently, we analysed both the structural gene and its upstream promoter region. The structural gene was identical to the wild-type sequence. However, Schwarz et al. suggested that the upstream region of *cfr*, composed of two ORFs [ORF1, 180 bp encoding a 59-amino acid protein; ORF2, 135 bp encoding a 44-amino acid protein (accession no. AJ249217)], is essential for florfenicol and chloramphenicol resistance (Schwarz et al. 2000). Genetic analysis of this region in the *cfr*-carrying plasmid from *S. simulans* SF3 revealed that the ORF1 amino acid sequence shared 98% identity with the wild-type, differing by a single substitution (L42F).

Interestingly, *S. simulans* SN1 and SF3 genomes differed by 31,481 SNPs by genome comparison.

*S. cohnii* SD1 carried the *cfr* gene on a 39,805-bp plasmid identical to plasmid unnamed1 from *S. cohnii* SDAQ-1, previously isolated from chicken in China (accession no. CP126541). In this plasmid, which did not belong to any known rep families, the gene was co-located with *fexA* within a Tn*558*-like transposon (accession no. AJ715531).

**Fig. 4:**
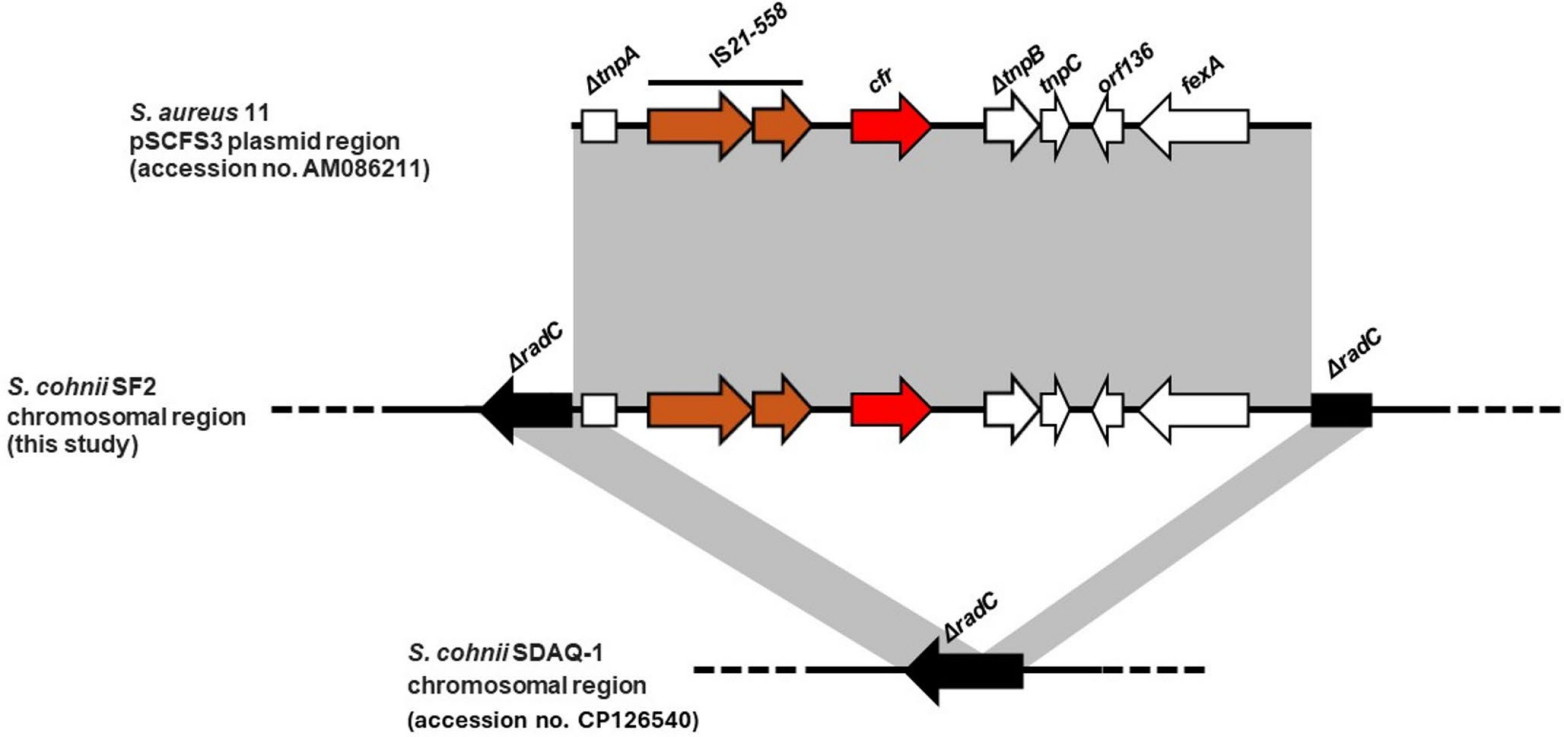
Schematic (to scale) comparative representation of the *S. cohnii* SF2 chromosomal regions and the *cfr* genetic context of plasmid pSCFS3. Open reading frames (ORFs) are depicted as arrows pointing in the direction of transcription; those common to *S. cohnii* chromosome region are in black, *cfr* in red; insertion sequences in brown, and ORFs of Tn*558*-like are in white. Δ symbol indicates a truncated gene. Grey areas between ORF maps denote > 90% DNA identity

In *S. cohnii* SF2, the *cfr* gene was located on a chromosomal region of 9,718 bp. Genome analysis revealed that it was carried by a Tn*558* transposon variant, similar to the corresponding DNA region previously described in the pSCFS3 plasmid from a *S. aureus* isolate of animal origin (accession no. AM086211) (Kehrenberg et al. 2006). This *cfr* genetic element also harboured the *fexA* gene, truncated *tnpA*–*tnpB* transposase genes, and the IS*21-558* insertion sequence. The chromosomal integration site of the *cfr*-containing Tn*558* variant was within the *radC* gene which encodes a DNA repair protein in the reference genome of *S. cohnii* SDAQ-1 strain (accession no. CP126540) (Fig. 4). Interestingly, *S. cohnii* SD1 and SF2 genomes differed by 17,031 SNPs by genome comparison.

**Fig. 5:**
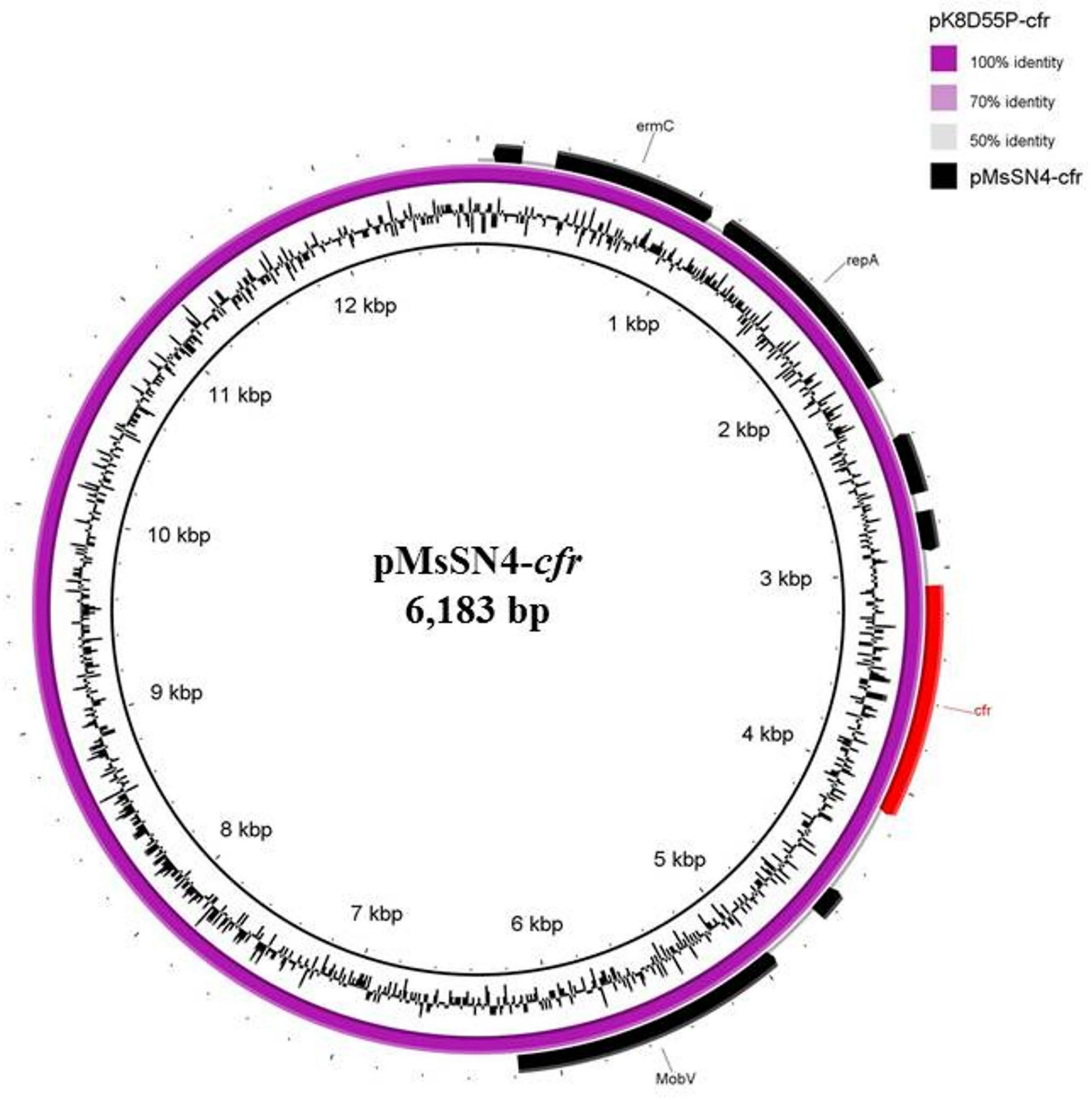
Circular map of the pMsSN4-*cfr* plasmid detected in *M. sciuri* SN4. The plasmid was aligned with the similar reported pK8D55P-*cfr* plasmid (accession no. CP065963) using BRIG software. Black arrows indicate the positions and orientations of genes; some antibiotic resistance determinants (red) and relevant genes described in this study are shown

Finally, *M. sciuri* SN4 harboured the *cfr* gene co-localized with the *erm*(C) gene (resistance to MLS_B_ antibiotics) on a small 6,183-bp novel plasmid (Rep1 replicon type), named pMsSN4-*cfr*. This plasmid showed 100% coverage and 96.47% nucleotide identity with the larger pK8D55P-*cfr* plasmid (12,724 bp; accession no. CP065963) from *M. sciuri* GDK8D55P, isolated from ducks in China (Schwarz et al. 2021) (Fig. 5, Table S4). Compared to pK8D55P-*cfr*, the new pMsSN4-*cfr* plasmid lacked a 6.5-kb region containing a gene encoding a Rep protein and *tet*(L) and *ant(4’)-Ia* genes.

## Discussion

Oxazolidinones are regarded as last-resort antimicrobials for the treatment of severe infections caused by multidrug-resistant Gram-positive bacteria (Schwarz et al. 2021; Brenciani et al. 2022).

Despite linezolid is not approved for use in food-producing animals, the increasing detection of oxazolidinone resistance genes in livestock-associated bacteria has raised significant concerns about the potential role of animals as reservoirs of clinically important antimicrobial resistance determinants (Schwarz et al. 2021; Brenciani et al. 2022).

To the best of our knowledge, this is the first report describing the presence of enterococci, staphylococci and *M. sciuri* carrying oxazolidinone resistance genes in wastewater collected from a swine slaughterhouse. These findings provide further evidence that slaughterhouses can serve as a hotspot for the dissemination of resistance genes within the food production environment. However, although the genomic characterization performed is comprehensive, the number of isolates analyzed is limited and reflects a specific time point; therefore, the reported prevalence may not fully capture potential temporal variability. The occurrence of *cfr*, *optrA* and *poxtA* genes in florfenicol-resistant animal enterococci, staphylococci, and in *M. sciuri* highlights once again the role of co-selection driven by the use of phenicols. Indeed, these genes not only confer resistance to linezolid, but also reduce susceptibility to florfenicol, which is extensively used in livestock production (Schwarz et al. 2021; Brenciani et al. 2022). Therefore, even antibiotic classes intended exclusively for veterinary use may indirectly contribute to the selection and persistence of resistance to last-line antimicrobials (Schwarz et al. 2021; Brenciani et al. 2022).

The distribution of oxazolidinone resistance genes across bacterial genera observed in this study is consistent with previous reports. The *cfr* gene was detected exclusively in staphylococci and *M. sciuri*, whereas enterococci harboured either *optrA* or *poxtA*. The *cfr* gene is well established as the primary determinant of linezolid resistance in staphylococci, whereas its role in resistance among enterococci remains poorly defined. Nevertheless, enterococci are considered important reservoirs of this resistance determinant (Schwarz et al. 2021; Brenciani et al. 2022; Desphande et al. 2018). In contrast, the *optrA* and *poxtA* genes are widely distributed among enterococci and, to a lesser extent, among staphylococci (Schwarz et al. 2021; Brenciani et al. 2022). This apparent host preference may reflect differences in the ecology and mobility of these genes; however, their localization on transferable elements underscores the potential for horizontal dissemination across bacterial populations (Schwarz et al. 2021; Brenciani et al. 2022).

Whole-genome sequencing revealed a remarkable diversity of genetic platforms associated with oxazolidinone resistance. In staphylococci, *cfr* was found on both novel and previously described plasmids, as well as integrated into chromosomal transposon-like structures (Schwarz et al. 2021; Brenciani et al. 2022). Notably, the detection of *cfr* within a Tn*558*-like element in *S. cohnii* SF2 further highlights the gene’s ability to integrate into diverse genomic contexts. This genetic plasticity may enhance the persistence of *cfr* even in the absence of selective pressure by linezolid, favoring its long-term maintenance and potential spread within bacterial populations (Schwarz et al. 2021; Brenciani et al. 2022).

A noteworthy finding was the detection of a *cfr*-positive *S. simulans* isolate that remained susceptible to linezolid despite carrying an intact *cfr* structural gene. In this isolate, the coding sequence was identical to the wild-type, whereas the upstream region showed a single amino acid substitution (L42F) in ORF1. Since the upstream region of *cfr* has been implicated in the expression of phenicol resistance (Schwarz et al. 2000), this observation may indicate that variation in regulatory elements contributes to the lack of phenotypic linezolid resistance. Similar *cfr*-positive but linezolid-susceptible isolates have previously been reported in both enterococci and staphylococci (Liu et al. 2014; Brenciani et al. 2016; Fioriti et al. 2020), supporting the notion that the presence of *cfr* alone is not always predictive of resistance. This genotype–phenotype discrepancy highlights the need for functional studies to better clarify the contribution of regulatory regions to *cfr* expression.

The identification of the *cfr* gene in *M. sciuri* SN4 on a small novel plasmid similar to pK8D55P-*cfr*, previously described in avian isolates, suggests the circulation of conserved plasmids among bacterial populations from different animal production systems. This finding reinforces the hypothesis that *M. sciuri* may serve as both a reservoir and vector of *cfr*, enabling its spread to staphylococci and potentially to more clinically relevant species (Li et al. 2016).

Among enterococci, the plasmid-mediated carriage of *optrA* or *poxtA* genes is of particular concern. The identification of two novel plasmids pEfmED1-*optrA* in *E. faecium* and pEhEM2-*poxtA* in *E. hirae* enhances our understanding of the genetic elements involved in oxazolidinone resistance. Furthermore, the detection in *E. durans* EF2 of *poxtA* within the Tn*6657* transposon (D’Andrea et al. 2019) — integrated into a multidrug resistance plasmid — underscores the risk of dissemination of oxazolidinone resistance through linked genetic elements. Such assemblies promote co-selection and may facilitate the spread of resistance across ecological and host boundaries.

The slaughterhouse wastewater environment likely provides favorable conditions for genetic exchange due to high bacterial densities (Cong et al. 2023).

Our findings raise serious public health concerns, as bacteria resistant to clinically relevant antibiotics — such as oxazolidinones — can colonize slaughterhouse workers, cross-contaminate carcasses during processing, and be released into the environment via wastewater treatment systems. The potential horizontal transfer of *cfr*, *optrA* and *poxtA* to human-adapted pathogens may compromise the clinical efficacy of oxazolidinones, limiting therapeutic options for serious infections due to Gram-positive bacteria. Moreover, the detection of shared plasmids and transposons in animal and human isolates suggests that extensive genetic exchange has occurred between these settings.

## Conclusion

This study reports the occurrence of transferable oxazolidinone resistance genes in enterococci, staphylococci and *M. sciuri* isolated from slaughterhouse wastewater, demonstrating that this environment can serve as a reservoir for such resistance determinants.

These findings underscore the urgent need for coordinated multisectoral action within a One Health framework — promoting the prudent use of antibiotics in livestock, improving animal husbandry practices, and implementing continuous surveillance measures — to mitigate the spread of bacteria resistant to last-resort antibiotics.

## Supplementary Information

Below is the link to the electronic supplementary material.

Supplementary Material 1

## Data Availability

The WGS data of *E. faecium* ED1 and EF1, *S. simulans* SN1 and SF3, *S. cohnii* SD1 and SF2, *E. durans* EF2, *E. hirae* EM2 and *M. sciuri* SN4 are available under the BioProject ID PRJNA1295837.
