# Supplementary material for: Occurrence of oxazolidinone resistance genes in enterococci, staphylococci, and Mammaliicoccus sciuri from swine slaughterhouse wastewater, Italy
Source: World J Microbiol Biotechnol. 2026 Apr 18;42(5):204. doi: 10.1007/s11274-026-04968-0 (PMC13090210; doi:10.1007/s11274-026-04968-0)
Supplement: Supplementary file 1 — Supplementary Material 1 [file 11274_2026_4968_MOESM1_ESM.docx]

**Table S1.** Amino acid sequence identities/similarities of putative proteins encoded by pEfmED1-*optrA* plasmid*.*

---------------------------------------------------------------------------------------------------------------------------------------------------------------------------------------------------------------------------------------------------- BLASTP analysis*a* Size --------------------------------------------------------------------------------------------------------------------------------------------

ORF Start Stop (amino Predicted function % Amino acid

(bp) (bp) acids) Most significant database match Accession no. identity (% amino

acid similarity)

----------------------------------------------------------------------------------------------------------------------------------------------------------------------------------------------------------------------------------------------------

*orf1* 1 273 91 Mobile element protein transposase [*Enterococcus faecium*] WP_343451037.1 100 (100)

*orf2* 461 940 159 hypothetical protein [*E. faecium*] WP_212463069.1 99 (99)

*orf3* 941 1581 213 hypothetical protein [*E. faecium*] WP_129204685.1 99 (99)

*orf4* 1596 2528 310 hypothetical protein [*E. faecium*] WP_234127551.1 99 (100)

*orf5* 2658 3281 207 recombinase family protein [*E. faecium*] WP_338360520.1 99 (99)

*orf6* 3311 3523 70 hypothetical protein [*E. faecium*] WP_369674971.1 96 (98)

*orf7* 3616 3906 96 hypothetical protein [*E. faecium*] UBL09799.1 97 (98)

*orf8* 4251 4574 107 UPF0758 family protein JAB domain-containing protein [*E. faecium*] WP_154731919.1 98 (99)

*orf9* 4688 5773 361 Transposase A from transposon Tn554 tyrosine-type recombinase/integrase [*Staphylococcus aureus*] WP_312013413.1 99 (100)

*orf10* 5774 7689 118 Transposase B from transposon Tn554 Helix-turn-helix transcriptional regulator [*C. difficile*] WP_330364231.1 100 (100)

*orf11* 7691 8056 639 Mobile element protein tyrosine-type recombinase/integrase [*S. aureus*] WP_312013629.1 99 (100)

*orf12* 8339 8755 138 Uncharacterized NAD(P)H oxidoreductase, NAD(P)H-dependent oxidoreductase, partial [*Lactococcus lactis*] WP_428282883.1 100 (100)

YdeQ/YrkL/YwrO family

*orf13* 8933 10360 475 Chloramphenicol/florfenicol resistance, chloramphenicol/florfenicol efflux MFS transporter FexA [*S. aureus*] WP_113556784.1 99 (99)

MFS efflux pump => FexA family

*orf14* 11223 11426 67 UPF0758 family protein JAB domain-containing protein [*E. faecalis*] WP_311054637.1 100 (100)

*orf15* 11433 12416 327 TnpY DUF3991 and TOPRIM domain-containing protein [*E. avium*] WP_311857339.1 99 (99)

*orf16* 12429 12599 56 hypothetical protein [*E. avium*] AXM43501.1 100 (100)

*orf17* 12905 17032 1375 helicase-related protein [*E. gallinarum*] WP_311828957.1 99 (100)

*orf16* 17131 17547 138 hypothetical protein [*Lactobacillales*] WP_204183926.1 99 (100)

*orf18* 17551 18261 236 DNA-binding protein SpoVG SpoVG family protein [*Lactobacillales*] WP_002415366.1 100 (100)

*orf19* 18567 20537 656 2,4-dienoyl-CoA reductase [NADPH] oxidoreductase [*Lactobacillales*] WP_002415367.1 100 (100)

*orf20* 20671 21825 384 helix-turn-helix domain-containing protein [*Streptococcus suis*] WP_024378354.1 99 (100)

*orf21* 22156 24123 655 ABC-F type ribosomal protection protein => ABC-F type ribosomal protection protein OptrA [*S. aureus*] WP_430727179.1 99 (99)

OptrA

*orf22* 25429 25989 186 23S Rrna 23S ribosomal RNA methyltransferase Erm, partial [*Enterococcus*] WP_219645655.1 100 (100) (adenine(2058) -N(6))-dimethyltransferase

=> Erm(A)

*orf23* 26380 27042 220 Mobile element protein class I SAM-dependent methyltransferase [*S. aureus*] WP_053015044.1 99 (99)

*orf24* 27637 28383 248 Methylase N-6 DNA methylase [*E. faecalis*] WP_240208236.1 99 (99)

*orf25* 28376 32713 1445 Type II restriction endonuclease Eco57I restriction-modification methylase domain-containing protein WP_010721122.1 100 (100)

[*E. faecium*]

*orf26* 33329 34654 441 ImpB/MucB/SamB family protein Y-family DNA polymerase [*S. aureus*] WP_002313331.1 96 (98)

*orf27* 34647 34997 116 DNA-directed RNA polymerase beta subunit hypothetical protein [*S. aureus*] WP_002321268.1 91 (94)

*orf28* 34998 35188 64 hypothetical protein [*E. faecium*] WP_098040870.1 95 (98)

*orf29* 35266 35628 120 hypothetical protein [*Enterococcus*] WP_002332907.1 100 (100)

*orf30* 35642 36448 268 partitioning protein ParA ParA family protein [*Enterococcus*] WP_002332906.1 100 (100)

*orf31* 36877 37920 347 Replication initiation protein A replication initiator protein A [*E. faecium*] WP_283592368.1 98 (98)

*orf32* 38795 39187 130 probable pheromone-responsive regulatory hypothetical protein [*Enterococcus*] WP_010722082.1 100 (100)

protein R

*orf33* 39754 39930 58 hypothetical protein [*E. faecium*] WP_415354006.1 98 (100)

*orf34* 39984 40160 58 hypothetical protein [*E. faecium*] UBL09760.1 100 (100)

*orf35* 40139 40369 76 hypothetical protein [*E. faecium*] WP_260466710.1 100 (100)

*orf36* 40393 40590 65 hypothetical protein [*E. faecium*] WP_002372456.1 98 (98)

*orf37* 40601 40873 90 hypothetical protein [*E. faecium*] WP_137240331.1 99 (100)

*orf38* 40984 41403 139 hypothetical protein [*E. faecium*] WP_098040866.1 99 (99)

*orf39* 41449 41691 80 hypothetical protein [*E. faecium*] WP_065772518.1 100 (100)

*orf40* 41914 42741 275 LysM peptidoglycan-binding domain-containing protein [*E. faecium*] WP_317914904.1 99 (99)

*orf41* 42942 46112 1056 SpaA isopeptide-forming pilin-related protein [*E. faecium*] WP_226397213.1 99 (100)

*orf42* 46161 47198 345 Sortase A, LPXTG specific class A sortase [*E. faecium*] WP_181727043.1 99 (99)

*orf43* 47285 48811 508 peptidoglycan DD-metalloendopeptidase family protein [*E. faecium*] WP_123067479.1 98 (98)

*orf44* 48990 49397 135 hypothetical protein [*E. faecium*] WP_002332892.1 100 (100)

*orf45* 49421 49873 150 hypothetical protein [*E. hirae*] WP_229065706.1 92 (92)

*orf46* 49912 50574 220 PcfB family protein [*E. faecium*] WP_248289684.1 95 (96)

*orf47* 50571 52445 624 TrsK-like protein VirD4-like conjugal transfer protein, CD1115 family, partial [*E. faecium*] WP_275350684.1 99 (99)

*orf48* 52462 52665 67 Permeases of the major facilitator hypothetical protein [*Enterococcus*] WP_086319127.1 100 (100)

superfamily

*orf49* 52692 53540 282 Tn5252, Orf23 hypothetical protein [*E. faecium*] WP_086319128.1 99 (99)

*orf50* 54124 56574 816 TrsE-like protein VirB4-like conjugal transfer ATPase, CD1110 family [*Enterococcus*] WP_098040861.1 100 (100)

*orf51* 56571 58418 615 CHAP domain-containing protein [*E. faecium*] HDL2739507.1 99 (99)

*orf52* 58439 59059 206 hypothetical protein [*E. faecium*] HCI1499083.1 100 (100)

*orf53* 59052 59426 124 thioredoxin domain-containing protein [*E. faecium*] WP_269021776.1 99 (100)

*orf54* 59426 59659 77 hypothetical protein [*E. faecium*] WP_283597224.1 100 (100)

*orf55* 59732 60118 128 hypothetical protein [*E. faecium*] WP_256925832.1 98 (100)

*orf56* 60139 61206 355 hypothetical protein [*E. faecium*] WP_002372491.1 96 (98)

*orf57* 61207 62785 528 hypothetical protein [*E. faecium*] WP_258390745.1 97 (98)

*orf58* 62804 63835 343 ArdC-like ssDNA-binding domain-containing protein [*Enterococcus*] WP_096638056.1 99 (100)

*orf59* 63917 64252 111 hypothetical protein [*E. faecium*] RBS25609.1 100 (100)

*orf60* 64273 64542 89 hypothetical protein [*E. faecium*] WP_201065248.1 98 (98)

*orf61* 64778 65191 137 plasmid mobilization protein [*Enterococcus*] WP_002332875.1 100 (100)

*orf62* 65192 67485 764 relaxase/mobilization nuclease domain-containing protein [*E. faecium*] WP_124018574.1 98 (98)

*orf63* 68012 68929 305 Mobile element protein IS256 family transposase, partial [*E. durans*] WP_161126709.1 99 (99)

----------------------------------------------------------------------------------------------------------------------------------------------------------------------------------------------------------------------------------------------

*^a^*For each ORF, only the most significant identity detected is listed

**Table S2.** Amino acid sequence identities/similarities of putative proteins encoded by pEhEM2-*poxtA* plasmid*.*

---------------------------------------------------------------------------------------------------------------------------------------------------------------------------------------------------------------------------------------------------- BLASTP analysis*a* Size --------------------------------------------------------------------------------------------------------------------------------------------

ORF Start Stop (amino Predicted function % Amino acid

(bp) (bp) acids) Most significant database match Accession no. identity (% amino

acid similarity)

----------------------------------------------------------------------------------------------------------------------------------------------------------------------------------------------------------------------------------------------------

*orf1* 155 1018 287 Zeta toxin DUF3991 domain-containing protein [*Streptococcus suis*] RRR58413.1 99 (98)

*orf2* 1020 1292 90 Epsilon antitoxin antitoxin [*Enterococcus faecalis*] WP_048901679.1 99 (98)

*orf3* 1310 1519 69 Tn916, transcriptional regulator, putative peptide-binding protein [*Staphylococcus aureus*] WP_111168123.1 97 (97)

*orf4* 1617 2513 298 ParA family protein [*S. aureus*] WP_115172198.1 98 (99)

*orf5* 3536 4339 267 Lincosamide nucleotidyltransferase lincosamide nucleotidyltransferase Lnu(B) [*S. aureus*] WP_113525274.1 100 (100)

=> Lnu(B)

*orf6* 4393 5877 494 ABC-F type ribosomal protection protein ABC-F type ribosomal protection protein Lsa(E) [*S. aureus*] WP_074371031.1 99 (99)

=> Lsa(E)

*orf7* 6320 6805 161 Site-specific recombinase recombinase zinc beta ribbon domain-containing protein [*E. faecalis*] WP_336603247.1 99 (100)

*orf8* 7268 8104 278 Mobile element protein IS3 family transposase [*S. aureus*] WP_176244455.1 99 (99)

*orf9* 8140 8430 96 Mobile element protein transposase [*E. faecium* EnGen0002] ELA78001.1 100 (100)

*orf10* 8527 8793 88 hypothetical protein [*Bacilli*] WP_240048080.1 100 (100)

*orf11* 8844 9134 96 RNA methyltransferase, TrmA family hypothetical protein [*S. suis*] HFI0355553.1 99 (100)

*orf12* 9313 10122 269 Aminoglycoside 9-nucleotidyltransferase aminoglycoside nucleotidyltransferase ANT(9) [*S. aureus*] WP_113619360.1 100 (100)

=> ANT(9)-I

*orf13* 10254 10781 175 Adenine phosphoribosyltransferase-like phosphoribosyltransferase family protein [*S. suis*] WP_105142556.1 100 (100)

protein

*orf14* 10825 11688 287 Aminoglycoside 6-nucleotidyltransferase aminoglycoside 6-adenylyltransferase [*S. aureus*] WP_165622132.1 99 (100)

=> ANT(6)-I

*orf15* 11721 12431 236 Methyltransferase, UbiE/COQ5 family methyltransferase domain-containing protein [*Enterococcus*] WP_166162137.1 99 (100)

*orf16* 12987 13724 245 23S rRNA 23S rRNA (adenine(2058)-N(6))-methyltransferase Erm(B) WP_172951588.1 99 (99)

(adenine(2058)-N(6))-dimethyltransferase [*Escherichia coli*]

=> Erm(B)

*orf17* 13981 14214 77 Tn916, transcriptional regulator, putative peptide-binding protein [*E. faecalis*] WP_411955684.1 99 (98)

*orf18* 14354 16498 714 DNA topoisomerase III type IA DNA topoisomerase [*Enterococcus*] WP_231477931.1 99 (99)

*orf19* 16498 17115 205 Resolvase recombinase family protein [*E. viikkiensis*] WP_311819720.1 96 (98)

*orf20* 17800 18156 118 Mobile element protein DDE-type integrase/transposase/recombinase [*S. aureus*] WP_000607310.1 100 (100)

*orf21* 18322 18735 137 replication control protein PrgN replication control protein PrgN family protein [*E. faecium* 13.SD.W.09] EPH64473.1 96 (97)

*orf22* 18745 20244 499 primase C-terminal domain-containing protein [*S. aureus*] WP_176244449.1 99 (99)

*orf23* 20795 21706 303 partitioning protein ParA ParA family protein [*E. faecium*] WP_194179438.1 99 (100)

*orf24* 21708 22079 123 hypothetical protein [*E. casseliflavus*] WP_142967633.1 98 (100)

*orf25* 22336 23022 228 Mobile element protein IS6-like element IS1216 family transposase [*Lactobacillales*] WP_060797357.1 99 (99)

*orf26* 23179 23556 125 hypothetical protein [*E. faecium*] WP_431415825.1 100 (100)

*orf27* 23693 23950 85 Mobile element protein transposase [*Bacillota*] WP_013330744.1 100 (100)

*orf28* 24160 24372 70 hypothetical protein [*Lactobacillus johnsonii*] WP_407325673.1 99 (100)

*orf29* 25061 26470 469 FexB chloramphenicol/florfenicol efflux MFS transporter FexB [*S. aureus*] WP_202978724.1 99 (99)

*orf30* 26800 27165 121 hypothetical protein [*E. faecium*] MBW4143076.1 100 (100)

*orf31* 27201 27887 228 Mobile element protein IS6-like element IS1216 family transposase [*Lactobacillales*] WP_060797357.1 99 (99)

*orf32* 28846 30474 542 PoxtA ABC-F type ribosomal protection protein PoxtA [*E. thailandicus*] WP_285702015.1 100 (100)

*orf33* 30522 31208 228 Mobile element protein IS6-like element IS1216 family transposase [*Lactobacillales*] WP_060797357.1 99 (99)

*orf34* 31336 32295 319 Integrase, catalytic region IS30-like element IS1252 family transposase [*E. faecium*] WP_176453292.1 99 (100)

*orf35* 33316 33921 190 Fic domain protein, HP1159 type recombinase family protein [*Enterococcus*] WP_135172343.1 96 (98)

*orf36* 34119 34805 228 Mobile element protein IS6-like element IS1216 family transposase [*Lactobacillales*] WP_060797357.1 99 (99)

*orf37* 34861 35316 151 Na+/H+ antiporter NapA cation:proton antiporter, partial [*E. faecium*] WP_311790009.1 98 (98)

*orf38* 35413 37449 678 Penicillin-binding protein 3 penicillin-binding protein PBP4(5) [*E. faecium*] WP_010732583.1 98 (98)

*orf39* 37580 38248 222 Cell envelope-associated transcriptional transcriptional regulator [*E. faecium*] MBK4757152.1 99 (99)

attenuator LytR-CpsA-Psr, subfamily F1

*orf40* 38320 39006 228 Mobile element protein IS6-like element IS1216 family transposase [*Lactobacillales*] WP_060797357.1 99 (99)

*orf41* 39636 40547 303 partitioning protein ParA ParA family protein [*E. faecium*] WP_194179438.1 99 (100)

*orf42* 41098 42597 499 primase C-terminal domain-containing protein [*S. aureus*] WP_176244449.1 99 (99)

*orf43* 42607 43020 137 replication control protein PrgN replication control protein PrgN family protein [*E. faecium* 13.SD.W.09] EPH64473.1 96 (97)

*orf44* 43186 43542 118 Mobile element protein DDE-type integrase/transposase/recombinase, partial [*S. aureus*] WP_000607310.1 100 (100)

*orf45* 44227 44844 205 Resolvase recombinase family protein [*E. viikkiensis*] WP_311819720.1 96 (98)

*orf46* 44844 45536 230 DNA topoisomerase III DNA topoisomerase [*S. aureus*] WP_430625310.1 99 (99)

*orf47* 45832 46329 165 Recombinase DNA recombinase [*E. faecium*] WP_222838994.1 100 (100)

*orf48* 46330 46746 138 Recombinase recombinase [*S. aureus*] WP_164096708.1 99 (99)

*orf49* 46748 48310 520 Site-specific recombinase recombinase family protein [*S. aureus*] WP_438873496.1 99 (99)

*orf50* 48692 49561 289 DNA polymerase, beta-like region nucleotidyltransferase domain-containing protein [*S. aureus*] WP_155560111.1 100 (100)

*orf51* 49542 50276 244 Methyltransferase, UbiE/COQ5 family class I SAM-dependent methyltransferase [*S. aureus*] WP_096827996.1 99 (99)

*orf52* 50309 51172 287 Aminoglycoside 6-nucleotidyltransferase aminoglycoside 6-adenylyltransferase [*S. aureus*] WP_165622132.1 99 (100)

=> ANT(6)-I

*orf53* 51216 51743 175 Adenine phosphoribosyltransferase-like phosphoribosyltransferase family protein, partial [*S. suis*] WP_105142556.1 100 (100)

protein

*orf54* 51875 52216 113 Aminoglycoside 9-nucleotidyltransferase nucleotidyltransferase domain-containing protein [*Clostridioides difficile*] HBH2017978.1 100 (100)

=> ANT(9)-I

*orf55* 52171 52683 170 Aminoglycoside 9-nucleotidyltransferase aminoglycoside nucleotidyltransferase ANT(9), partial [*S. aureus*] WP_113619360.1 100 (100)

=> ANT(9)-I

*orf56* 52862 53152 96 RNA methyltransferase, TrmA family hypothetical protein [*S. suis*] HFI0355553.1 99 (100)

*orf57* 53203 53661 152 hypothetical protein [*S. aureus*] WP_275273309.1 100 (100)

*orf58* 53729 54307 192 Site-specific recombinase recombinase zinc beta ribbon domain-containing protein [*S. aureus*] WP_318010004.1 99 (99)

*orf59* 54749 56233 494 ABC-F type ribosomal protection protein ABC-F type ribosomal protection protein Lsa(E) [*S. aureus*] WP_074371031.1 99 (99)

=> Lsa(E)

*orf60* 56287 57090 267 Lincosamide nucleotidyltransferase, Lnu(B) lincosamide nucleotidyltransferase Lnu(B), partial [*S. aureus*] WP_113525274.1 100 (100)

*orf61* 57204 58124 306 Integrase, catalytic region IS30-like element IS1252 family transposase [*E. faecium*] WP_176453292.1 99 (100)

*orf62* 58252 58962 236 Mobile element protein IS6-like element IS1216 family transposase [*Lactobacillales*] WP_060797357.1 96 (96)

*orf63* 59431 60384 317 ParA family protein [*E. faecium*] WP_086324098.1 98 (99)

*orf64* 60996 62489 496 primase C-terminal domain-containing protein [*S. aureus*] WP_242414046.1 99 (99)

*orf65* 62624 62920 98 replication control protein PrgN type III secretion system protein PrgN [*E. durans*] WP_311931566.1 99 (98)

*orf66* 63021 63872 283 hypothetical protein [*E. faecalis*] WP_048962559.1 99 (99)

*orf67* 64532 65218 228 Mobile element protein IS6-like element IS1216 family transposase [*Lactobacillales*] WP_060797357.1 99 (99)

*orf68* 65252 65575 107 Truncated replication protein for pUB110 protein rep [*E. faecium*] WP_425461167.1 100 (100)

plasmid

*orf69* 65819 67081 420 Plasmid recombination enzyme MobV family relaxase [*S. aureus*] WP_279524512.1 99 (100)

*orf70* 67212 67595 127 hypothetical protein [*E. faecium*] WP_408640007.1 96 (96)

*orf71* 67644 69020 458 Tetracycline resistance, MFS efflux pump tetracycline efflux MFS transporter Tet(L) [*S. aureus*] WP_165622105.1 99 (100)

=> Tet(L)

Δ*orf72* 69214 69531 105 Tetracycline resistance, ribosomal protection hypothetical protein [*E. coli*] WP_407224459.1 100 (100)

type => Tet(M)

Δ*orf73* 69525 70565 346 Tetracycline resistance, ribosomal protection TetM/TetW/TetO/TetS family tetracycline resistance ribosomal WP_231418372.1 100 (100)

type => Tet(M) protection protein, partial [*E. faecalis*]

Δ*orf74* 70555 71130 191 Tetracycline resistance, ribosomal protection TetM/TetW/TetO/TetS family tetracycline resistance ribosomal WP_231418372.1 99 (100)

type => Tet(M) protection protein, partial [*E. faecalis*]

*orf75* 71507 72439 310 CD3337/EF1877 family mobilome membrane protein [*S. anginosus*] WP_080654274.1 99 (99)

*orf76* 72436 73437 333 lipoprotein, NLP/P60 family bifunctional lytic transglycosylase/C40 family peptidase [*S. aureus*] WP_031797198.1 99 (99)

*orf77* 73434 75611 725 putative membrane protein CD3337/EF1877 family mobilome membrane protein WP_203290550.1 98 (98)

[*Metamycoplasma hominis*]

*orf78* 75614 78061 815 TcpE family conjugal transfer membrane protein [*S. pyogenes*] WP_281727386.1 100 (100)

*orf79* 78045 78551 168 Tn916, hypothetical protein conjugal transfer protein [*S. aureus*] WP_431197279.1 99 (99)

*orf80* 78526 79023 165 Antirestriction protein conjugative transposon protein [*Ureaplasma urealyticum* EDX54014.1 100 (100)

serovar 9 str. ATCC 33175]

*orf81* 79140 79361 73 Conjugation related protein hypothetical protein [*E. cecorum*] WP_441652859.1 100 (100)

*orf82* 79404 80609 401 Tn916, transcriptional regulator, putative MobT family relaxase [*Streptococcus*] WP_049519213.1 100 (100)

*orf83* 80632 80784 50 conjugative transposon hypothetical protein conjugal transfer protein [*S. pneumoniae*] WP_088783750.1 100 (100)

*orf84* 80787 82172 461 FtsK/SpoIIIE family protein FtsK/SpoIIIE domain-containing protein [*S. aureus*] WP_031910377.1 99 (99)

*orf85* 82201 82584 127 YdcP family protein [*E. faecium*] NTP54671.1 100 (100)

*orf86* 82603 82917 104 YdcP family protein [*S. aureus*] HHR7028671.1 100 (100)

----------------------------------------------------------------------------------------------------------------------------------------------------------------------------------------------------------------------------------------------

*^a^*For each ORF, only the most significant identity detected is listed.

ΔTrunacted ORFs.

**Table S3.** Amino acid sequence identities/similarities of putative proteins encoded by pSsSN1-*cfr* plasmid.

---------------------------------------------------------------------------------------------------------------------------------------------------------------------------------------------------------------------------------------------------- BLASTP analysis*a* Size --------------------------------------------------------------------------------------------------------------------------------------------

ORF Start Stop (amino Predicted function % Amino acid

(bp) (bp) acids) Most significant database match Accession no. identity (% amino

acid similarity)

----------------------------------------------------------------------------------------------------------------------------------------------------------------------------------------------------------------------------------------------------

*orf1* 154 375 73 Hypothetical protein [*Staphylococcus aureus*] WP_197854954.1 100 (100)

*orf2* 377 1156 259 ParA family protein [*S. aureus*] WP_254231378.1 100 (100)

*orf3* 1333 1944 203 hypothetical protein [*S. aureus*] WP_111739705.1 100 (100)

*orf4* 1960 3048 362 CHAP domain-containing protein [*S. aureus*] WP_373697126.1 100 (100)

*orf5* 3049 3562 175 hypothetical protein [*S. pseudointermedius*] WP_096636928.1 100 (100)

*orf6* 3574 5574 666 CD3337/EF1877 family mobilome membrane protein [*S. aureus*] WP_095284924.1 100 (100)

*orf7* 5588 6184 198 hypothetical protein [*S. aureus*] WP_095284925.1 100 (100)

*orf8* 6185 8769 862 ATP-binding protein [*S. aureus*] WP_275276605.1 100 (100)

*orf9* 8773 9231 152 Hypothetical protein [*S. aureus*] WP_115287857.1 100 (100)

*orf10* 9247 9540 97 hypothetical protein [*S. agnetis*] WP_252566117.1 100 (100)

*orf11* 9552 10715 387 conjugal transfer protein [*S. agnetis*] WP_252566114.1 100 (100)

*orf12* 10740 11600 286 replication-relaxation family protein [*S. aureus*] WP_070703014.1 100 (100)

*orf13* 11654 14392 912 Hypothetical protein [*S. aureus*] WP_301555467.1 100 (100)

*orf14* 15408 16982 524 primase C-terminal domain-containing protein [*S. aureus*] WP_172687460.1 100 (100)

*orf15* 17377 17619 80 helix-turn-helix domain-containing protein [*S. aureus*] WP_095324641.1 100 (100)

*orf16* 17620 18194 191 DNA-invertase recombinase family protein [*S. aureus*] WP_240022750.1 96 (98)

*orf17* 18544 19551 335 Hypothetical protein [*S. aureus*] WP_084984958.1 100(100)

*orf18* 19555 20268 237 Hypothetical protein [*S. aureus*] WP_031873336.1 100 (100)

*orf19* 20738 22063 441 TcaA NTF2-like domain-containing protein [*S. aureus*] WP_231414884.1 100 (100)

*orf20* 22082 22777 231 Hypothetical protein [*S. aureus*] WP_142301160.1 98 (99)

*orf21* 22794 23519 241 Hypothetical protein [*S. aureus*] WP_031905641.1 100 (100)

*orf22* 23597 23989 130 YolD-like family protein [*S. aureus*] WP_001632630.1 100 (100)

*orf23* 24012 24260 82 Hypothetical protein [*S. aureus*] WP_001632631.1 100 (100)

*orf24* 24418 24564 48 DNA repair protein RadC JAB domain-containing protein [*S. aureus*] WP_261976678.1 100 (100)

*orf25* 25427 26854 475 Chloramphenicol/florfenicol resistance, MFS chloramphenicol/florfenicol efflux MFS transporter FexA [*S. aureus*] WP_113556784.1 99 (99)

efflux pump => FexA family

*orf26* 27167 27841 224 Mobile element protein IS6-like element IS257 family transposase [*S. aureus*] WP_187417939.1 99 (100)

*orf27* 27885 28343 152 Replication protein protein rep, partial [*S. aureus*] WP_029052284.1 98 (100)

*orf28* 28343 28525 60 hypothetical protein [*Mammaliicoccus vitulinus*] WP_107559459.1 97 (98)

*orf29* 29079 29900 273 Ribosomal RNA large subunit putative RNA methyltransferase, partial [*S. aureus*] WP_257241795.1 100 (100)

methyltransferase A

*orf30* 30179 30691 170 Replication protein replication protein [*S. aureus*] CAD24833.1 100 (100)

*orf31* 30733 31407 224 Mobile element protein IS6-like element IS257 family transposase [*S. aureus*] WP_187417939.1 100 (100)

*orf32* 31423 32784 453 ABC-F type ribosomal protection protein, ABC-F type ribosomal protection protein Lsa(B) [*S. aureus*] WP_371090356.1 100 (100)

Lsa(B)

*orf33* 33135 33314 59 hypothetical protein [*Mammaliicoccus sciuri*] WP_032489810.1 98 (98)

*orf34* 33468 34517 349 23S rRNA (adenine(2503)-C(8))- 23S rRNA (adenine(2503)-C(8))-methyltransferase Cfr [*Escherichia coli*] WP_338846666.1 99 (99)

methyltransferase, Cfr

*orf35* 35318 35866 182 recombinase family protein [*Salinicoccus jeotgali*] WP_344701653.1 94 (97)

*orf36* 35899 36204 101 helix-turn-helix domain-containing protein [*Staphylococcus*] WP_032495386.1 100 (100)

*orf37* 36246 36920 224 Mobile element protein IS6-like element IS257 family transposase [*S. aureus*] WP_187417939.1 99 (99)

*orf38* 36921 37751 284 Truncated replication protein for pUB110 protein rep [*S. aureus*] WP_234722905.1 97 (97)

plasmid

*orf39* 37920 38681 253 Aminoglycoside 4'-nucleotidyltransferase, aminoglycoside O-nucleotidyltransferase ANT(4')-Ia [*S. aureus*] QCJ22238.1 99 (100)

ANT(4')-Ia

*orf40* 38988 40364 458 Tetracycline resistance, MFS efflux pump, tetracycline efflux MFS transporter Tet(L) [*S. aureus*] WP_165622105.1 99 (100)

Tet(L)

*orf41* 40647 41138 163 Dihydrofolate reductase DfrD/DfrG/DfrK family trimethoprim-resistant dihydrofolate WP_031913333.1 99 (99)

reductase [*S. aureus*]

*orf42* 41589 41942 117 MazG nucleotide pyrophosphohydrolase domain-containing protein WP_241373577.1 96 (95)

[*S. aureus*]

*orf43* 42538 43800 420 Plasmid recombination enzyme MobV family relaxase [*S. aureus*] WP_279524512.1 96 (98)

*orf44* 44172 44324 50 Truncated replication protein for pUB110 protein rep [*Staphylococcus*] WP_049948302.1 100 (100)

plasmid

*orf45* 44352 45026 224 Mobile element protein IS6-like element IS257 family transposase [*S. aureus*] WP_187417939.1 99 (99)

*orf46* 45119 45334 71 hypothetical protein [*S. aureus*] WP_063280111.1 99 (100)

*orf47* 45357 45734 125 hypothetical protein [*S. aureus*] WP_095324631.1 98 (99)

*orf48* 45908 46105 65 Phage protein hypothetical protein [*S. aureus*] MEZ2067969.1 100 (100)

*orf49* 46152 46310 52 hypothetical protein [*S. aureus*] WP_172693015.1 98 (98)

*orf50* 46307 46501 64 hypothetical protein [*S. aureus*] WP_172693016.1 95 (95)

*orf51* 46641 46988 115 hypothetical protein [*S. aureus*] WP_238518297.1 98 (99)

*orf52* 47001 47861 286 DNA/RNA non-specific endonuclease [*S. aureus*] WP_165619710.1 94 (97)

*orf53* 47879 48847 322 Extracellular protein DUF1002 domain-containing protein [*S. aureus*] WP_095341267.1 100 (100)

*orf54* 48869 49279 136 hypothetical protein [*S. aureus*] WP_231415063.1 96 (98)

*orf55* 49727 50056 109 DNA repair protein RadC JAB domain-containing protein [*S. aureus*] WP_165621528.1 98 (100)

*orf56* 50170 51255 361 Transposase A from transposon Tn*554* tyrosine-type recombinase/integrase [*S. aureus*] WP_312013413.1 99 (100)

*orf57* 51252 53171 639 Transposase B from transposon Tn*554* tyrosine-type recombinase/integrase [*S. aureus*] WP_312013629.1 99 (100)

*orf58* 53173 53538 121 Mobile element protein DUF6262 family protein [*S. aureus*] WP_191835658.1 99 (99)

*orf59* 53821 54210 129 Uncharacterized NAD(P)H oxidoreductase, NAD(P)H-dependent oxidoreductase [*S. aureus*] WP_267428928.1 100 (100)

YdeQ/YrkL/YwrO family

*orf60* 54226 54900 224 Mobile element protein IS6-like element IS257 family transposase [*S. aureus*] WP_187417939.1 99 (99)

--------------------------------------------------------------------------------------------------------------------------------------------------------------------------------------------------------------------------------------------

*^a^*For each ORF, only the most significant identity detected is listed

**Table S4.** Amino acid sequence identities/similarities of putative proteins encoded by pMsSN4-*cfr* plasmid.

---------------------------------------------------------------------------------------------------------------------------------------------------------------------------------------------------------------------------------------------------- BLASTP analysis*a* Size ----------------------------------------------------------------------------------------------------------------------------------------------------------------------------------------------------

ORF Start Stop (amino Predicted function % Amino acid

(bp) (bp) acids) Most significant database match Accession no. identity (% amino

acid similarity)

----------------------------------------------------------------------------------------------------------------------------------------------------------------------------------------------------------------------------------------------------

*orf1* 68 199 43 Hypothetical protein [*Enterococcus faecium*] UBL10235.1 98 (100)

*orf2* 351 1085 244 Ribosomal RNA adenine dimethylases 23S rRNA methyltransferase Erm(C) [*Staphylococcus*] WP_181187314.1 95 (97)

*orf3* 2141 1158 327 Replication protein Protein rep, partial [*Enterococcus faecalis*] WP_262252954.1 100 (100)

*orf4* 2659 2384 91 Hypothetical protein, partial [*Klebsiella pneumoniae*] WP_443093606.1 98 (98)

*orf5* 2742 2921 59 Hypothetical protein [*Mammaliicoccus sciuri*] WP_032489810.1 98 (98)

*orf6* 3075 4124 349 23S rRNA methyltransferase, Cfr 23S rRNA methyltransferase Cfr [*Enterococcus pseudoavium*] WP_311797642.1 99 (99)

*orf7* 6183 4933 416 MobV family relaxase Plasmid recombination protein [*Planomicrobium okeanokoites*] TAA65602.1 93 (95)

---------------------------------------------------------------------------------------------------------------------------------------------------------------------------------------------------------------------------------------------------
